# The recombinant zoster vaccine induces trained immunity in monocytes through persistent downregulation of TGFβ

**DOI:** 10.1371/journal.ppat.1013759

**Published:** 2025-12-05

**Authors:** Michael J. Johnson, Megan Crotteau, Debashis Ghosh, Thao Vu, Luke Trinity, Radu Marches, Duygu Ucar, Myron J. Levin, Adriana Weinberg

**Affiliations:** 1 Department of Pediatrics, University of Colorado School of Medicine, University of Colorado Denver, Anschutz Medical Campus, Aurora, Colorado, United States of America; 2 Department of Biostatistics and Informatics, Colorado School of Public Health, University of Colorado Anschutz Medical Campus, Aurora, Colorado, United States of America; 3 The Jackson Laboratory for Genomic Medicine, Farmington, Connecticut, United States of America; 4 Department of Medicine, University of Colorado School of Medicine, University of Colorado Denver, Anschutz Medical Campus, Aurora, Colorado, United States of America; 5 Department of Pathology, University of School of Medicine, University of Colorado Denver, Anschutz Medical Campus, Aurora, Colorado, United States of America; State University of New York Upstate Medical University, UNITED STATES OF AMERICA

## Abstract

Older adults have decreased vaccine efficacy, but the adjuvanted recombinant VZV-gE zoster vaccine (RZV) is highly efficacious. We investigated memory-like innate immune responses after RZV and after the zoster vaccine live (ZVL), which is much less efficacious. RZV increased NK, monocyte, and DC activation in response to in vitro VZV-gE stimulation for up to 5 years post-vaccination, while ZVL increased only DC responses to VZV for up to 90 days. In purified monocyte and NK cell cocultures, RZV recipients showed increased responses to VZV-gE, HCMV and HSV antigenic stimulation post-vaccination. ATAC-seq analysis of purified monocytes revealed decreased accessibility in areas of the *TGFβ1* gene. scRNA-seq and immunoproteomics confirmed decreased *TGFβ1* transcription and translation, respectively. Exogenous supplementation and inhibition of TGFβ1 modulated in vitro monocyte responses to VZV-gE. In conclusion, RZV generated homologous (VZV-gE) and heterologous (HCMV, HSV) trained immunity in monocytes through genomic repression of the regulatory cytokine TGFβ-1. Cytokine modulation may represent a novel mechanism of generating trained immunity in myeloid cells.

## Author summary

Older adults have decreased vaccine efficacy, but the adjuvanted recombinant varicella zoster virus (VZV)-gE zoster vaccine (RZV; Shingrix™) is highly efficacious. We investigated memory-like innate immune responses after RZV and after the zoster vaccine live (ZVL; Zostavax™), which is much less efficacious than RZV. We found that RZV increased the functionality of several innate immune cell subsets against VZV-gE other herpesviruses. The increase in functionality was associated with decreased production of the inhibitory cytokine *TGFβ1,* which may have resulted from decreased ability to use the *TGFβ1* gene as a template for the synthesis of its product. We concluded that RZV generated homologous (VZV-gE) and heterologous (other herpesviruses) memory-like responses in innate immune cell subsets through genomic repression of the regulatory cytokine TGFβ-1.

## Introduction

Herpes zoster (HZ) is a severe disease caused by the reactivation of latent varicella-zoster virus (VZV). Asymptomatic VZV reactivations are not uncommon, but viral replication is generally controlled by the host immune response [1–4]. Clinical manifestations of HZ ensue when the immune system fails to contain the viral spread. HZ is most frequent in people with decreased cell-mediated immunity (CMI), including those with congenital immunodeficiencies, AIDS, iatrogenic immunosuppression, and older adults with immunosenescence [5–8]. Moreover, the risk of HZ is not increased by selective antibody deficiency, nor is it mitigated by antibody supplementation [9,10]. Thus, protection against HZ is deemed to be primarily mediated by CMI. Additional evidence links the incidence of HZ to a decrease in VZV-specific Th1 responses [11]. However, the exact mechanisms that limit viral spread during VZV reactivation have not been defined. In addition to adaptive immunity, protection against HZ may involve innate CMI [12–14].

Innate immune cells are rapidly deployed as the first line of defense against pathogens. Moreover, innate immune cells are critical for the development of adaptive immunity. Subsequently, innate and adaptive immune cells may establish feedback mechanisms that boost each other [15–21]. Recent evidence revealed that innate immune cells develop memory-like responses [17,21–34]. Unlike conventional T and B cells, innate immune cells either lack antigen-specific receptors or have receptors with limited capacity for recombination. Instead, the development of memory-like responses in innate immune cells relies on epigenetic modifications and clonal expansions [32,35]. The best studied viral infection controlled by memory-like innate immunity is human cytomegalovirus (HCMV), which, like VZV, is a member of the Herpesviridae family that is defined by a life cycle consisting of acute infection, followed by latency and reactivation. HCMV-specific adaptive NK cells, which are stimulated by HCMV peptides expressed in the context of HLA class I E, have high in vitro cytolytic capacity against HCMV-infected cells and confer in vivo protection in a murine HCMV infection model and in human transplant recipients [35–39]. These adaptive NK cells have well-characterized epigenetic modifications underlying their memory-like immune responses [35,40,41]. VZV-specific adaptive NK cells have also been described in HZ skin lesions [42]. Another set of innate immune cells with memory-like immune responses against HCMV are the Vδ2^-^ and Vγ9^-^ γδ T cells [43–45]. These cells undergo limited T-cell receptor (TCR) rearrangement, which confers HCMV specificity and the ability to control HCMV reactivations [43–46]. Monocytes and dendritic cells (DC) can also develop memory-like responses termed trained immunity [32–34]. Epigenetic modifications associated with trained immunity were described in the context of severe COVID-19 [47] infections in circulating monocytes and hematopoietic stem cells. This trained immunity is maintained through epigenetic modifications of hematopoietic stem and progenitor cells [47,48].

Vaccines are available to protect against many infections, including HZ, but people at the highest risk of developing these infections tend to have poor responses to vaccines. This is exemplified by the Zoster Vaccine Live (ZVL), which confers 70% protection against HZ in adults 50–59 years of age, 64% in adults aged 60–69 years, 41% in adults aged 70–79 years and no protection in adults aged ≥80 years in the first year after vaccination [49,50]. Moreover, the protection conferred by ZVL completely wanes 10 years after vaccination. In contrast, a recombinant zoster vaccine (RZV) containing the VZV glycoprotein E (gE) combined with the potent adjuvant AS01B changed this paradigm. RZV achieved 3-year efficacy >90% and 10-year efficacy >70% against HZ in healthy adults ≥50 years of age. Efficacy and persistence were minimally affected by the age of the vaccinee [51–53]. We previously showed that RZV generates higher levels than ZVL of gE- and VZV-specific CD4 + Th1 memory, CD8 + cytotoxic lymphocytes, and antibodies [54–57]. Other investigators also showed higher Th17 responses after RZV than after ZVL [58]. However, adaptive T cell immune responses measured after administration of live attenuated viral vaccines typically require a week to reach peak activity [59]. Thus, innate immune responses may be essential for initial control of infection. The ability of RZV to generate memory-like immune responses in innate cells has not been studied, although other live-attenuated, adjuvanted, and mRNA vaccines are considered to have this capability [27,60–64].

Using blood samples collected in a randomized double-blind study entitled “Comparison of Live Herpes Zoster Vaccine with a Recombinant Vaccine in 50- 59 and 70- 85 year olds” (NCT 02114333), we showed VZV-gE-specific innate immune responses in peripheral blood mononuclear cells (PBMC) after RZV administration, but not after ZVL. We further demonstrated that increased responses in monocytes, DC and NK cells persist for up to 5 years after RZV administration and that genomic repression of the *TGFβ1* regulatory cytokine likely contributes to monocyte trained immunity.

## Methods

### Ethics statement

The study was approved by the Colorado Multiple Institutional Review Board. All participants provided signed informed consent.

#### Study design.

This study used samples from a subset of 27 participants enrolled in a randomized study comparing RZV and ZVL immune responses (NCT 02114333; **S1 Fig**) [55]. For the initial experiments, we randomly selected 10 RZV and 10 ZVL participants from NCT 02114333. PBMC from additional participants were added to subsequent experiments to ensure that adequate numbers of samples were tested at each time point for the 5-year time course and for each condition in the depletion and reconstitution, and genomic experiments.

#### Flow cytometry assays.

PBMC were cryopreserved for viability within 8 h of collection and stored at ≤-150°C until use. To assess T cell activation in freshly thawed PBMC, we used co-expression of CD38 and HLA-DR, markers commonly used for this purpose and extensively studied on cryopreserved PBMC [65,66]. To detect antigen-presenting cell (APC) activation in freshly thawed and stimulated PBMC, we tested CD40, CD80, CD83, CD86 and PDL-1 markers and found that CD83 and PDL-1 were the most discriminative markers between stimulated and unstimulated conditions, which prompted their inclusion in the final staining panel. PBMC were thawed, counted, and promptly stained with two different panels of mAbs. Both staining panels included an initial phosphate-buffered saline (PBS) wash and viability staining with Zombie Yellow Viability dye (BioLegend). Cells were subsequently washed with PBS supplemented with 1% bovine serum albumin (BSA; Sigma; A9576-50ML) and incubated at room temperature with Human TruStain FcX (BioLegend). In the APC and B-cell panel, the cells were next washed and stained with mAbs anti-CD14 conjugated with Ax488, anti-CD83 PE, anti-CD141 PE-Dazzle, anti-CD123 PerCPCy5.5, anti-CD56 PE-Cy7, anti-CD1c Ax700, anti-HLA-DR APCcy7, anti-PDL-1 BV421, anti-CD3 PE-Cy7, anti-CD19 APC, and True-Stain Monocyte Blocker (BioLegend) and Brilliant Stain Buffer (BD Biosciences). For the T-cell panel, the cells were stained with mAbs anti-CD16 FITC, anti-CCR7 PE-Dazzle, anti-CD8 PerCPcy5.5, anti-CD56 PE-Cy7, anti-HLA-DR APC, anti-CD38 BV421, anti-γδ TCR PE, anti-CD3 Ax700, and anti-CD45RO APC-H7. Both panels were washed and resuspended in PBS + 1% paraformaldehyde before acquisition on a Gallios instrument (Beckman Coulter). The data were analyzed with Flow Jo (BD Biosciences). The gating strategy is shown in **S2 Fig**, and the key resource table is displayed in **S1 Table**.

For responses to in vitro stimulation, pre-optimization experiments for the T cell panel showed that IFNγ, TNFα, IL10, GranzB, CD134 and PD-1 were selective for conventional T cells and/or there was poor discrimination between stimulated samples and unstimulated controls, whereas CD137 and CD25 yielded a strong activation signal on both NK and T cells. For APC activation, PDL-1 and CD83 were the most discriminative between stimulated and unstimulated conditions as mentioned above. Cryopreserved PBMC were thawed, washed, counted, and resuspended at 5 × 10^6^ cells/mL in AIM-V medium (Gibco; 12-055-091). VZV vOKA virus, recombinant VZV-gE (VZV-rgE; gift from GSK), VZV-gE overlapping peptides (VZV-gE pp; gift from GSK), R848 (Mabtech), and rhIL2 (Sigma) were added to PBMC at final concentrations of 60,000 plaque-forming units/ml, 5 µg/mL, 2.5 µg/mL, 0.05 µg/mL, and 95.5 ng/mL, respectively. The antigen concentrations were designed for optimal stimulation, whereas the R848 and rhIL2 concentrations were suboptimal to allow the detection of increased activation post-vaccination. After a 20-h incubation at 37°C in a CO_2_-enriched humidified atmosphere, the cells were washed with PBS, stained with Zombie yellow viability dye (BioLegend), and analyzed using the following staining panels: 1) cells in the 1-year kinetic APC panel were stained as above; 2) cells in the 1-year kinetics T-cell panel were stained for CD16 FITC, CCR7 PE-Dazzle, CD8 PerCP-Cy5.5, CD56 PE-Cy7, CD137 APC, CD45RO BV421 (BioLegend), γδ TCR PE, CD3 Ax700, CD45RO BV421, and CD25 APC-Cy7 (BD Biosciences); and 3) cells for the 5-year kinetics were stained with PDL1 BV421, CD14 PE-CF594, HLA-DR APC-H7, CD3 Ax700, CD20 Ax700, CD25 BV785, CD137 PE, CD16 FITC, CD56 PE-Cy7, and CD19 Ax700.

Panels 1 and 2 were acquired on a Gallios instrument, and panel 3 was acquired on a NovoCyte Quanteon instrument (Agilent). Data analysis was performed using FlowJo (BD Biosciences). Gating Strategy is shown in **S3 Fig**.

#### ATAC-seq assays.

PBMC cryopreserved before and 90 days after vaccination were thawed, washed, and counted. The cells were stained with Zombie Aqua (Biolegend), incubated with Human TruStain FcX (Biolegend), and surface-stained with CD16 FITC (Biolegend), CD14 PE-CF594 (BD Biosciences), CD56 PE-Cy7 (Biolegend), CD3 Ax700 (BD Biosciences), CD19 Ax700 (Biolegend), CD20 Ax700 (Biolegend), and HLA-DR APCH7 (BD Biosciences). The cells were then resuspended in buffer containing PBS, 1 mM EDTA (Corning), 25 mM HEPES (Corning), and 1% BSA (Sigma) and filtered before sorting on a MoFlo Astrios Cell Sorter (Beckman Coulter). Monocytes were identified as CD14 positive, HLA-DR positive, and CD3, CD56, CD19, and CD20 negative. NK cells were identified as CD14-, CD3-, CD19-, and CD20-negative and CD16- and/or CD56-positive. Before starting the ATAC-seq library prep, the cell numbers were normalized to 100,000 per sample for monocytes and 150,000 per sample for NK. An ATAC-Seq Kit (Active Motif) was then used and the protocol followed according to the manufacturer’s instructions. Sequencing of the libraries was conducted with a NovaSeq 6000 (Illumina), and 40 million read pairs were recorded per sample.

#### Homologous and heterologous immune responses of purified monocytes and NK cells.

PBMC cryopreserved before and 90 days after vaccination were thawed and washed before cell separation. Monocytes were enriched with the EasySep Human Monocyte Enrichment Kit (Stemcell Technologies), and NK cells were enriched with the EasySep Human NK Cell Enrichment Kit (Stemcell Technologies) according to the manufacturer’s instructions. Then, 30,000 purified monocytes or 30,000 monocytes combined with 15,000 NK cells were cultured in AIM-V media supplemented with either VZV-rgE at 5 µg/ml, HCMV and herpes simplex virus (HSV) UV-inactivated cell lysates prepared as previously described [67], or unstimulated control. Purified pre-vaccination monocyte and NK cell cocultures stimulated with VZV-rgE were also treated with the TGFβ1 inhibitor LY2109761 (LY; Sigma‒Aldrich) at 0.1 µg/ml, and day 90 post-vaccination cocultures with recombinant human TGF-beta 1 protein (R&D Systems; 240-B-002/CF) at a concentration of 0.1 ng/ml in the presence of rgE or unstimulated control. All cultures were incubated for 48 h and stained with Zombie Aqua, PDL-1 BV421 (BD Biosciences), CD69 BV786 (BioLegend), CD137 PE (BioLegend), CD16 FITC (BioLegend), CD14 PE-CF594 (BD Biosciences), CD56 PE-Cy7 (BioLegend), CD3 Ax700 (BD Biosciences), CD19 Ax700 (BioLegend), CD20 Ax700 (BioLegend), HLA-DR APCH7 (BD Biosciences), and True-Stain Monocyte Blocker. Samples were run on the NovoCyte Quanteon, and FlowJo was used for analysis.

#### scRNA-seq assays.

Thawed cryopreserved PBMC were suspended in RPMI medium 1640 supplemented with l-glutamine, sodium pyruvate, HEPES buffer, penicillin-streptomycin and 10% v/v FBS prewarmed at 37°C and spun down. Cell pellets were washed once with PBS, spun down and then resuspended in PBS containing 0.04% bovine serum albumin. Cell viability was assessed using a Luna-FX7 (Logos Biosystems) with Acridine Orange/Propidium Iodide (Logos Biosystems). We performed nuclei extraction using a 2 min lysis incubation following the manufacturer’s protocol (CG000365). A total of 15,400 nuclei were loaded onto one lane of a Chip J (10X Genomics) to achieve a recovery of ~ 10,000 nuclei/sample. Single-nuclei capture, barcoding and library preparation for ATAC-seq and gene expression were performed using the 10x Chromium multiome assay chemistry following the manufacturer’s protocol (CG000338). cDNA and libraries were checked for quality using an Agilent 4200 Tapestation and Thermo Fisher Qubit Fluorometer, quantified by KAPA quantitative PCR and sequenced on an Illumina NovaSeq X + 10B flowcell, using 100-cycle (GEX) and 200-cycle (ATAC) for gene expression and ATACseq respectively, and targeting 8.8 to 10K barcoded nuclei/sample with a minimum sequencing depth of 70,000 read pairs per cell.

#### TGFβ1 concentration in culture supernatants.

Supernatants were collected from pre-vaccination and day 90 monocyte cultures after 48-h stimulation with rgE (5µg/ml) or medium control and flash frozen. On the day of the assay, the supernatants were thawed and TGFβ1 was activated using the Sample Activation Kit 1 (R&D Systems) as per the instructions from the TGFβ1 ELISA kit (RayBiotech). The activated supernatants were then diluted 10-fold with diluent B before starting the assay. The protocol from the TGFβ1 ELISA kit was followed and absorbance was read using a Multiskan FC reader (Thermo Scientific) with SkanIt 7.0.2 software (Thermo Scientific). Prism 10.1.1 (GraphPad) was used to calculate final concentrations.

### Statistical and bioinformatics analysis

#### ATACseq analysis.

FASTQ files were uploaded to the Galaxy platform [68] for analysis. FastQC was used to investigate read quality, and Cutadapt was used to cut out the adapter sequences. Reads were then mapped to the human reference genome (hg38) using Bowtie2. Uninformative reads were filtered out based on read mapping quality (<30 on the phred quality scale), proper pairing, mitochondrial reads, and duplicate reads. The insert size distribution was then checked to verify the expected nucleosomal pattern. Files were converted from BAM to BED format, and peak calling was performed using MACS2 [69]. The bedgraph output files from the peak calling were then converted to BigWig format for easier visualization of the genome. DiffBind was used to produce a normalized read count matrix, and the matrix was read into R [70] for differential chromatin accessibility analysis using DESeq2 [71]. FDR was used to adjust for multiple comparisons, and modifications were considered significant if the p value was < 0.1. The significantly modified genes were annotated using the biomaRt package [72], and PathfindR [73] was used for pathway analysis.

#### scRNA-seq analysis.

Illumina base call files for all libraries were converted to FASTQs using bcl2fastq v2.20.0.422 (Illumina) and aligned to the GRCh38 reference assembly with v32 annotations from GENCODE (10x Genomics GRCh38 reference 2020-A) using Cell Ranger arc v2.0.1 (10x Genomics). The gene-cell expression matrices were corrected for ambient RNA contamination using SoupX [74] prior to downstream analyses. Vireo [75] and Cellsnp [76] were used to demultiplex each pooled sample (i.e., one male and one female) using genotype information. Additionally, expression of y-chromosome genes [77] and/or XIST expression resolved cell assignment to male vs. female donors in pooled samples. Count matrices of PBMCs from four males and four females were obtained. Doublets were manually confirmed via expression of PBMC lineage markers before being removed from downstream analyses. The following quality control criteria were used for cell exclusion: (1) cells with fewer than 100 genes or more than 4000 genes by counts (2) cells with mitochondrial reads greater than 30% and (3) cells with more than 12,500 total counts. Genes that were present in less than three cells were also removed leaving 32,606 genes detected across all cells. Filtered gene expression matrices were merged and processed using the Scanpy toolkit (version 1.10.3) [78]. Read counts were normalized to 10,000 using the “normalize total” followed by “log1p” transformation. Genes with the highest normalized dispersion were identified using the “highly_variable_genes” function. Principal Component Analysis (PCA) was performed using the “pca” function. Harmony [79] was applied using the “harmony_integrate” function from Scanpy to correct batch effects across samples.

30 Principal Components (PCs) were used to calculate nearest neighbors via the “neighbors” function which were used to perform uniform manifold approximation and projection (UMAP) via the “umap” function. Initial clusters were determined using the “leiden” function for all PBMCs. Multiple rounds of marker identification, cell type annotation, and manual inspection and doublet removal, were performed to annotate PBMC subsets, resulting in six annotated PBMC lineages (see **S10A Fig**) from 86,550 cells across 16 samples (n = 8 donors, two timepoints, ~ 5000 cells per donor/timepoint). Here, we focused on transcriptomic analysis of the myeloid compartment, identifying eight myeloid cell subtypes (see **S10B Fig**).

Raw RNA counts were aggregated into counts per million (CPM) across myeloid cell types using the function “ADPBulk” from adpbulk package (GitHub: noamteyssier, 2021, adpbulk). Raw counts were first converted to a “DGEList” object, and transformed to normalized matrices using the “calcNormFactors” and “cpm” functions in edgeR [80]. Differentially expressed gene (DEG) analysis was performed using generalized linear models, employing the default normalization method based on the trimmed mean of M-values (TMM). P-values were adjusted using Benjamini–Hochberg correction for multiple comparisons and genes with an adjusted P-value (FDR) < 0.1 and log_2_ (fold change)> ±0.25 were selected as DEGs.

#### Flow cytometry and ELISA data analysis.

For experiments that included >2 timepoints, Friedman test with FDR adjustment was used to determine differences between timepoints using Prism 10.1.1 for MacOS software (GraphPad). Significance was defined by FDR p < 0.1. Experiments that involved 2 timepoints, pre- and post-vaccination, were analyzed using the Wilcoxon matched-pairs signed rank test using Prism 10.1.1 for MacOS and significance was defined by p < 0.05. Spearman correlation analysis was performed using Prism 10.1.1 for MacOS and significance was defined by p < 0.05.

## Results

### Demographic characteristics of the study population

This study used samples from 10 ZVL and 37 RZV recipients enrolled in a previous study [55]. A schematic representation of the parent study design is shown in **S1 Fig**. Participants who contributed samples to this study had a mean (range) age of 67 (50–79) years. Of the 47 total participants, 25 were female and 45 were White Non-Hispanic without appreciable differences between the vaccine groups.

### The frequency of activated innate and adaptive immune cells in peripheral blood increases only after RZV administration

**Fig 1:**
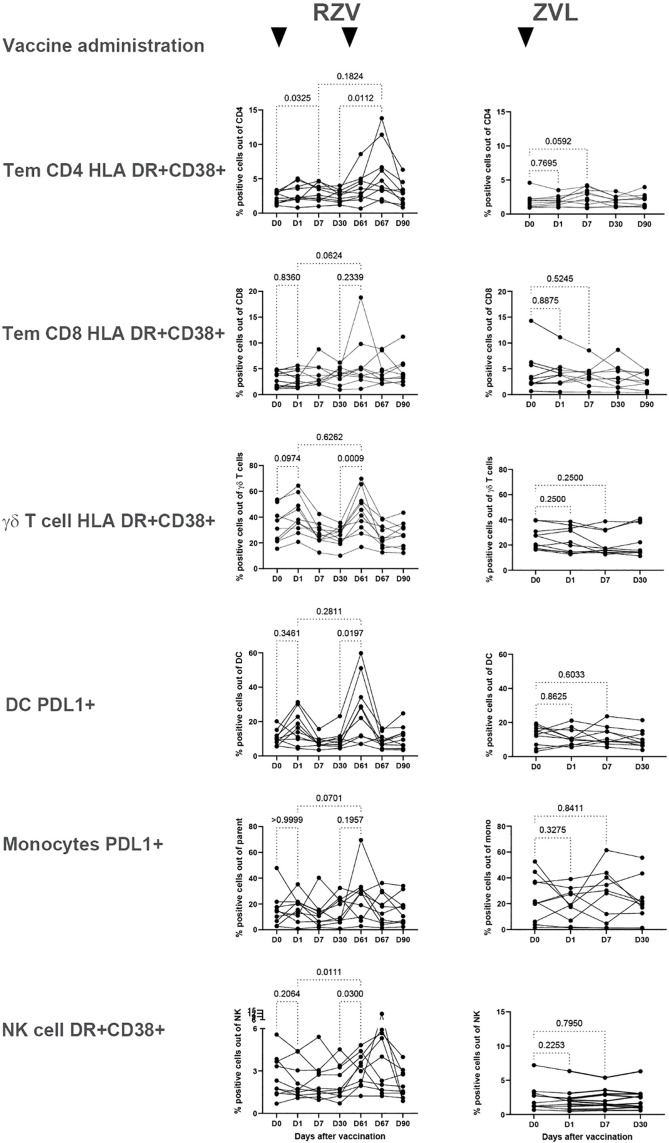
Peripheral blood innate and adaptive T cell responses to zoster vaccines. Data were derived from 10 RZV recipients who received 2 doses of vaccine on Days 0 and 60 and 10 ZVL recipients who received a single dose of vaccine on Day 0 as indicated by top arrows. Demographic characteristics of the participants are presented in S2 Table. The graphs show the frequencies of activated innate cells and conventional T cells in peripheral blood from pre-vaccination (D0) and up to 30 days after the last dose of vaccine for each participant. p values shown on the graphs were generated using Friedman test for repeated measures with FDR correction for multiple comparisons. RZV participants showed transient increases of activated immune cells in blood at 1, 7, 61 and/or 67 after the 1^st^ dose of vaccine. ZVL administration generated increases of activated CD4 + effector memory T cells (Tem; CD45RO+CCR7-) only.

To determine the innate immune responses to zoster vaccines, we measured the activation of monocytes, DC, NK and γδ T cells in peripheral blood up to 30 days after each dose of vaccine in 10 ZVL and 10 RZV recipients whose demographic characteristics are presented in **S2 Table**. For reference, we analyzed activation of conventional CD4+ and CD8 + T cells and B cells representing adaptive immunity. Based on pre-optimization experiments, activation of NK and T cells was measured by co-expression of HLA-DR and CD38 (Gating strategy shown in **S2 Fig**) and of B cells, monocytes, and DC by expression of PDL-1 and CD83. Since PDL-1 and CD83 expressions significantly correlated with each other (**S4 Fig**) and PDL-1 was previously described as a marker of trained immunity in monocytes and DC [81,82], only PDL-1 expression was used in the statistical analyses and subsequent experiments. The analysis showed activation of both innate cells and conventional T cells in peripheral blood post-vaccination but with diverging kinetics. CD4 + effector memory T cells (Tem; CD45RO+CCR7-) significantly increased after each dose of RZV- administered on Days 0 and 60 with peaks on Days 7 and 67 (FDR p ≤ 0.03; **Fig 1**). ZVL recipients showed significant increases in activated CD4 + Tem on Day 7 (FDR p = 0.06). In contrast, activated innate immune cells increased in peripheral blood on Days 1 and/or 61 post-RZV compared to pre-vaccination levels. Activated γδ T cells reached significantly higher levels both on Days 1 and 61 post-RZV (FDR p < 0.1) compared to pre-dose 1 (Day 0) and pre-dose 2 measured on Day 30, respectively, without a significant difference between the two post-vaccination time points; activated DC had a significant increase on Day 61 (FDR p = 0.02) compared to pre-dose 2; monocytes, like CD8 + Tem, did not significantly increase on Days 1 or 61 compared with pre-dose 1 or 2, respectively, but significantly increased from Day 1 to Day 61 (FDR p ≤ 0.07); NK cells significantly increased on Day 61 compared with Day 1 and with pre-dose 2 (FDR p ≤ 0.03). We did not detect increased activation in the peripheral blood of the innate cells in the 10 ZVL recipients (**Fig 1**), or of B cells in either vaccine group (not depicted).

Innate immune cells may be activated by cytokines secreted by conventional CD4 + T cells - a process known as bystander activation [83,84]. However, differences in the kinetics of activation between CD4 + Tem and innate immune cells argued against bystander activation. Correlation analyses revealed significant associations only between activated CD4 + Tem or activated total CD4 + T cells and activated γδ T cells on Days 1 and 61 (rho ≥ 0.9 and p ≤ 0.001 by Spearman correlation analysis; **S5 Fig**). The activation of other innate immune cells did not correlate with either CD4 + T-cell activation (**S5 Fig**).

### Parallel development of memory-like immune responses in innate and adaptive immune cells after RZV administration

**Fig 2:**
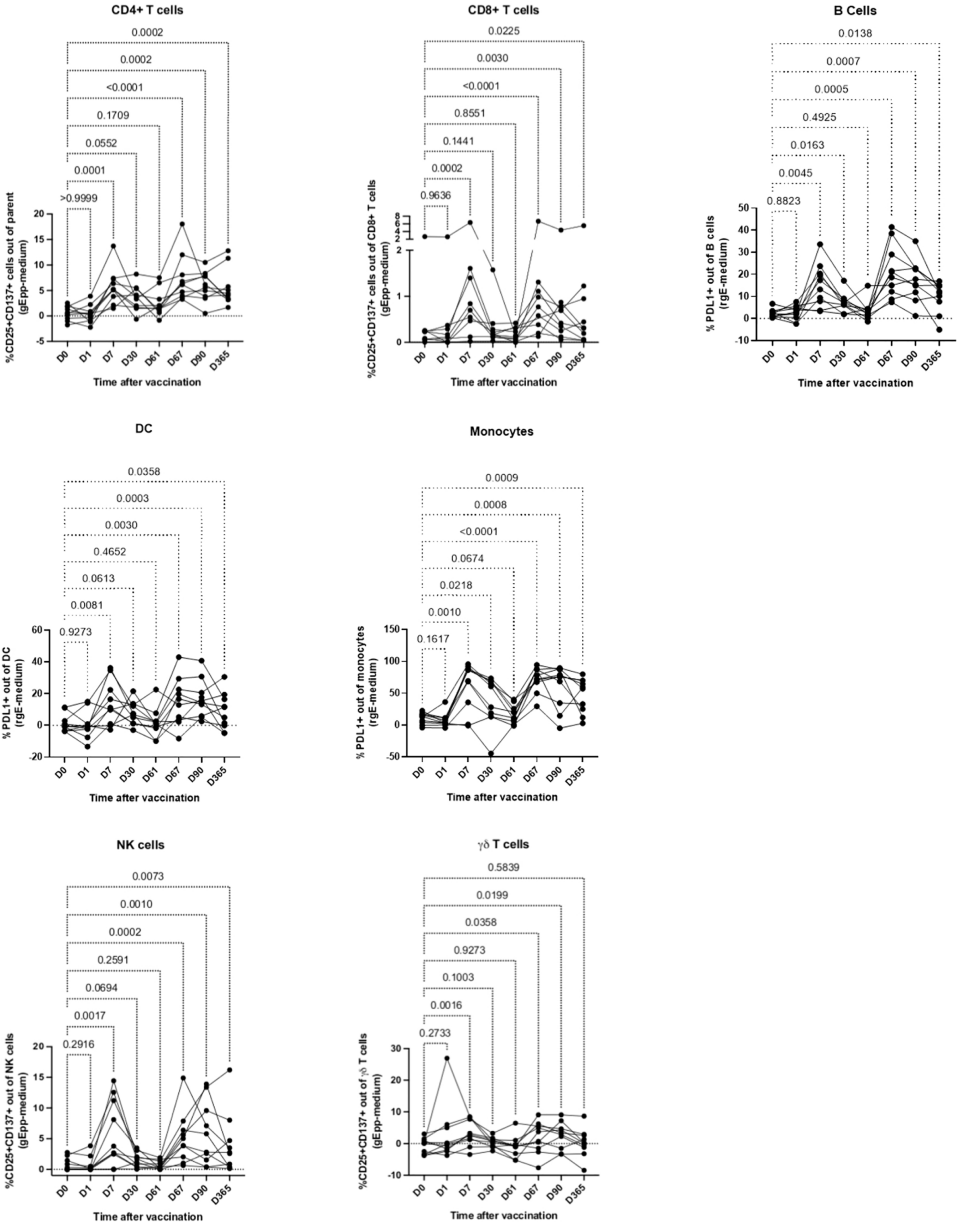
Innate and adaptive immune cell activation in response to ex vivo VZV-gE stimulation in RZV recipients. Data were derived from 10 participants who received 2 doses of RZV on Days 0 and 60. PBMC were stimulated overnight with VZV-gE peptides (gE pp) or recombinant VZV-gE (rgE) and medium control as indicated on the graphs. Demographic characteristics of the participants are presented in S2 Table. The graphs show the frequency of activated cells in antigen-stimulated conditions after subtraction of medium control from pre-vaccination up to 1 year post-vaccination in each participant. p values for the comparison of pre-vaccination with post-vaccination results were calculated using Friedman test for repeated measures with FDR correction for multiple comparisons. Responses of adaptive and innate immune cells followed similar trajectories except for γδ T cells. See also **S6 Fig** for responses in ZVL recipients.

To test the hypothesis that RZV generates memory-like immune responses in innate immune cells, we stimulated ex vivo PBMC from 10 RZV recipients with gE pp or rgE and medium unstimulated control and from 10 ZVL recipients with whole virus VZV and medium. The stimulation was performed in PBMC obtained from all study visits in the first year after vaccination. Activation was measured by the expression of PDL-1 on B cells, DC, and monocytes and by the co-expression of CD25 and CD137 on NK, γδ, and conventional T cells in antigen-stimulated conditions after the subtraction of unstimulated controls based on pre-optimization experiments described in the Methods section. The gating strategy is shown in **S3 Fig**. Compared with pre-vaccination, RZV recipients showed activation of B cells, DC, monocytes, NK, and CD4+ and CD8 + conventional T cells on Day 7, which rapidly decreased to Day 30 (**Fig 2**). Activation increased again on Day 67 (7 days after the 2^nd^ dose of vaccine) followed by a very gradual decrease to Days 90 and 365 and remaining above pre-vaccination levels. Activated γδT cells also increased above pre-vaccination levels in response to antigenic stimulation on Days 7, 67 and 90 but returned to pre-vaccination levels on Day 365. ZVL recipients showed increased activation of the adaptive B cells and conventional CD4 + T cells up to Day 365, of DC up to Day 90, and of conventional CD8 + T cells on Day 30 (**S6 Fig**).

Correlation analysis of the proportions of CD4 + T cells activated by VZV-gE ex vivo in RZV recipients with activated B cells, CD8 + T cells, DC, monocytes, NK and γδ T cells revealed significant associations of CD4 + T cells with CD8 + T cells, γδ, and NK cells on Days 0 and 7 (**S7 Fig**). Correlations between CD4+ and CD8 + T-cell activation were also noted on Days 90 and 365 (**S7 Fig**). The lack of significant associations between CD4 + T cells and innate cells after the 2^nd^ dose of vaccine suggested that bystander activation did not play a role in the activation of these cells after the 2^nd^ dose of the vaccine.

### Memory-like immune responses of DC, monocytes and NK cells persist for up to 5 years after RZV administration

**Fig 3:**
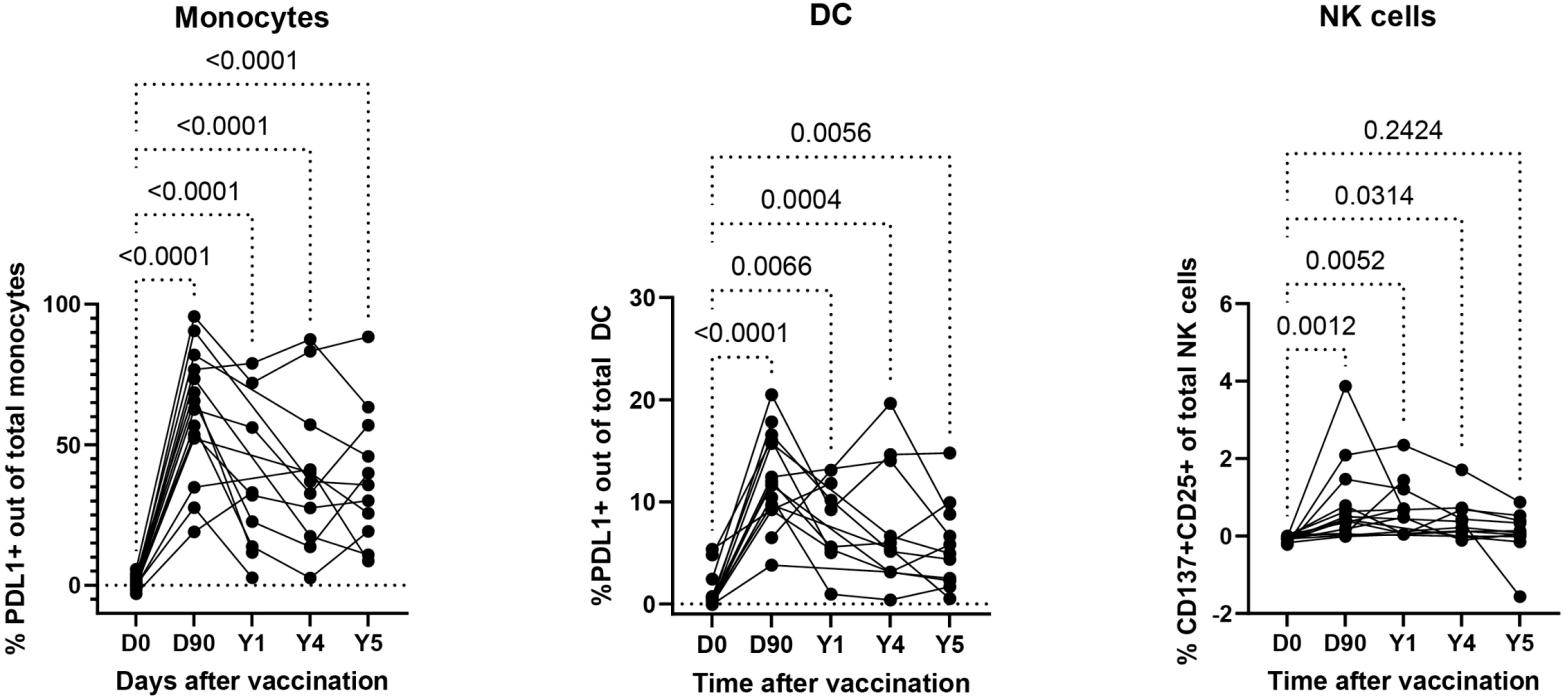
5-year persistence of innate immune responses to VZV-gE after RZV administration. Data were derived from 14 RZV recipients who received 2 doses of vaccine at Days 0 and 60. Demographic characteristics of the participants are presented in S3 Table. The graphs show the frequencies of activated cells in VZV-rgE-stimulated conditions after subtraction of medium control. p values were calculated using mixed effects analysis and FDR correction for multiple comparisons.

To investigate the persistence of innate immune cell responses generated by RZV, we stimulated ex vivo PBMC from 14 RZV recipients at 0, 3, 12, 48, and 60 months post-vaccination with VZV-rgE and unstimulated medium control. Demographic characteristics of the participants are presented in **S3 Table**. Activation of monocytes and DC was measured by expression of PDL-1, which was previously described as a marker of trained immunity on myeloid cells [81,82]. DC and monocyte responses to VZV-rgE persisted at significant levels above pre-vaccination for 5 years and that NK cells persisted for 4 years (**Fig 3**). Of note, one participant had an outlier result on Year 5 in the NK analysis, without which the difference between Year 5 and pre-vaccination would have been significant. However, following the administration of RZV, we did not observe responses to the nonspecific stimulators R848 or rhIL2 used at suboptimal doses to identify vaccine-induced increased responses (**S8 Fig**).

### Purified monocytes and NK cells from RZV recipients develop homologous and heterologous memory-like immune responses

**Fig 4:**
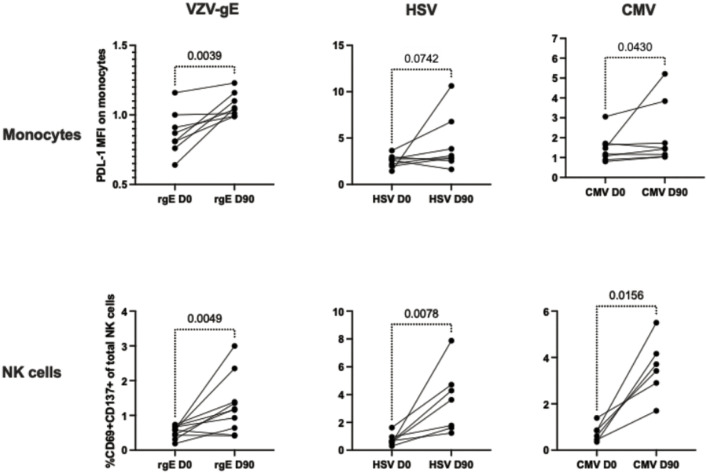
Homologous (VZV-gE) and heterologous (HSV, HCMV) Monocyte and NK memory-like immune responses after RZV administration. Data were derived from 10 RZV recipients. Demographic characteristics of the participants are presented in S4 Table. Monocytes and NK cells were purified using magnetic bead separation kits, combined at a ratio of 2:1, and incubated overnight with VZV-rgE, HSV lysate or HCMV lysate, and medium control. Graphs show ratios of antigen-stimulated over background control results before (D0) and after (D90) vaccination. p values were calculated by Wilcoxon matched-pairs signed rank test.

**Fig 5:**
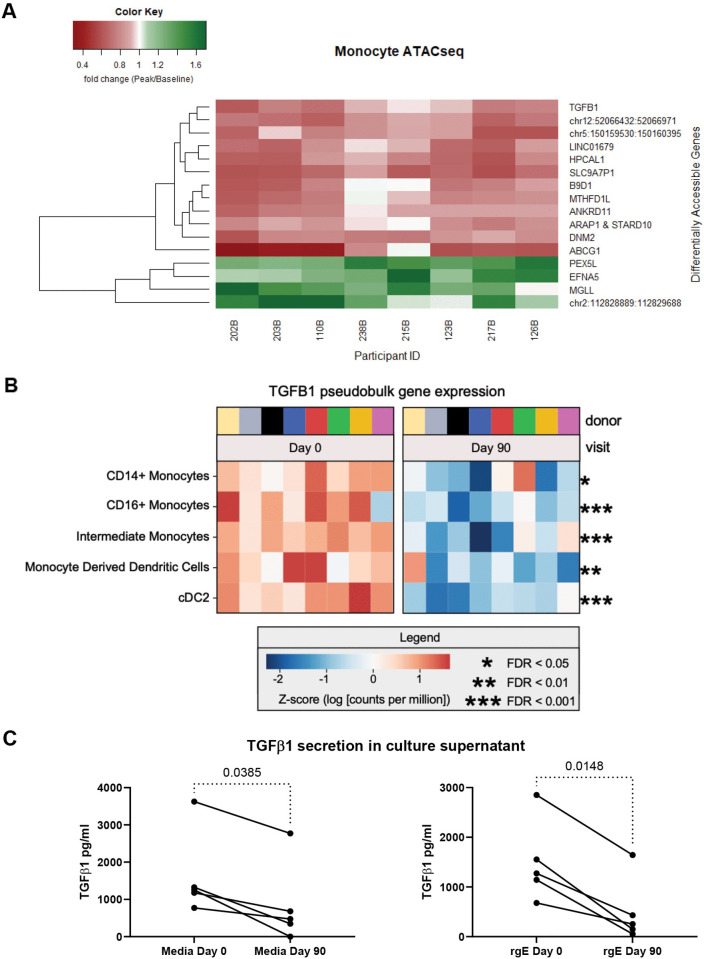
Genomic downregulation of TGF β1 production in monocytes obtained after RZV administration. Panel A. The heatmap shows 16 genes on the y axis whose accessibility significantly changed between Days 0 and 90 after vaccination (FDR p < 0.1) in 8 participants shown on the x axis. Note decreased accessibility of the *TGFβ1* gene. Panel B. The heatmap shows *TGFβ1* pseudobulk gene expression. Y-axis indicates myeloid lineage cell type (also see S10B Fig). Heatmap displays the z-score of log_10_ [counts per million] for pseudobulked *TGFβ1* transcripts. Each donor/timepoint is a separate column, with timepoints grouped together (left: day 0; right: day 90). Panel C. Decreased secretion of TGFβ1 in culture supernatants of purified monocytes obtained on Days 90 after RZV administration compared with pre-vaccination (Day 0). The graphs show TGFβ1 concentration in paired samples obtained from 5 RZV recipients. The x axes show the culture conditions, and the y axes show the concentrations of TGFβ1 measured by ELISA. p values were calculated by Friedman paired analyses with FDR correction for multiple comparisons.

To confirm that responses to VZV-gE detected in innate immune cells did not depend on T cell activation, we performed in vitro stimulation experiments using purified monocytes and NK cell cocultures. Monocytes and NK cells were isolated from PBMC obtained on Days 0 and 90 from 10 RZV recipients by negative depletion of T cells, B cells, granulocytes, dendritic cells and erythroid cells using magnetic beads. We then combined monocytes and NK cells from the same donor at a ratio of 2:1, roughly replicating their ratios in PBMC. The resulting cocultures had < 2% T-cell contamination. Cocultures were stimulated with VZV-rgE to detect homologous memory-like immune responses and with HCMV lysate and HSV lysate to detect heterologous responses. We tested samples from a mixture of HCMV- and HSV-seropositive and seronegative participants. Activation was measured by the expression of PDL-1 on monocytes and the co-expression of CD69 and CD137 on NK cells based on pre-optimization experiments described in the Methods section. After subtraction of medium control-stimulated results from antigen-stimulated conditions, we found significant increases in activation from pre- to post-vaccination, both in NK cells and monocytes, in response to all antigens (**Fig 4**). Notably, purified monocytes in isolation did not respond to ex vivo VZV-rgE stimulation (**Fig 5C**), suggesting that cross talk between monocytes and NK cells was essential for activation. To verify the interactions of NK cells and monocytes, we analyzed the doublets in the monocyte and NK cell cocultures representing attached cells and showed significantly higher co-expression of CD14, CD16, and CD56 compared to singlets, suggesting that the doublets were enriched in coupled monocytes and NK cells (**S9 Fig**).

### Epigenetic, transcriptional, and translational modifications associated with RZV administration

Monocytes and other innate immune cells can develop memory-like immune responses through epigenetic modifications [32]. We investigated epigenetic modifications and confirmed our findings at transcriptional and translational using PBMC from 20 RZV recipients (demographics in S5 Table). We performed bulk ATAC-seq on CD14 + CD16- monocytes sorted from PBMC obtained before and 90 days after the first dose of vaccine from 8 RZV recipients. We found 16 loci that were differentially accessible between pre- and post-vaccination timepoints (FDR-adjusted p value <0.1) (Fig 5A). Among these loci associated with *TGFβ1*, *DNAM2*, and *ARAP1* exhibited decreased accessibility, while loci associated with *MGLL* and *EFNA5* exhibited increased accessibility post-vaccination compared with pre-vaccination.

Although differentially accessible peaks associated with *TGFβ1* were distal (28 KB from transcription start site), given the importance of *TGFβ1* as a regulatory cytokine, we further investigated the transcription and translation of *TGFβ1* in monocytes obtained from pre-vaccination and Day 90 post-vaccination timepoints. Using scRNA-seq, we found downregulation of *TGFβ1* transcripts in CD16 + , CD14 + , and intermediate monocytes on Day 90 post-vaccination compared to pre-vaccination (FDR p ≤ 0.02; Figs 5C and S10). Notably, we also found decreased *TGFβ1* transcription in monocyte-derived DC and cDC2 (FDR p ≤ 0.02; Figs 5C and S10). To investigate *TGFβ1* translation, we cultured purified monocytes overnight in the presence of ZV-rgE or medium control and found significantly lower TGFβ1 concentrations in culture supernatants post-vaccination compared with pre-vaccination in both conditions (p ≤ 0.04; Fig 5C).

ATAC-seq analysis of purified NK cells from 10 participants revealed differential expression of 13 genes from pre- to postimmunization **S11 Fig**). However, we did not identify any genes or pathways associated with immune responses.

### TGF β1 modulates ex vivo monocyte responses to VZV-rgE in RZV recipients

**Fig 6:**
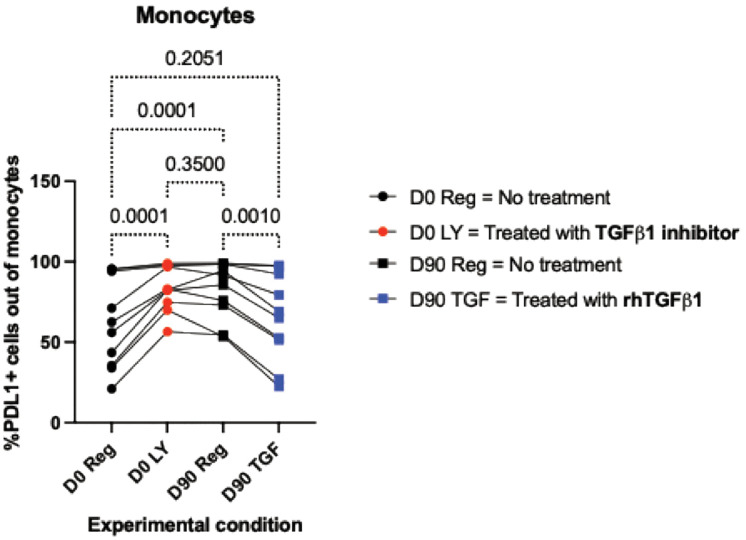
Effect of ex vivo treatment with rhTGFβ1 or a TGFβ1 inhibitor on monocyte responses to rgE stimulation. Monocytes and NK cells purified from PBMC collected from 10 RZV recipients (demographics in S4 Table) before vaccination (D0) and 90 days post-vaccination (D90) were combined ex vivo and stimulated with VZV-rgE. A subset of D0 monocyte & NK cocultures was also treated with the TGFβ1 inhibitor LY (D0 LY), and a subset of D90 cocultures were supplemented with rhTGFβ1. The graph shows individual data points, means and p values calculated by Friedman test for repeated measures with FDR correction. LY treatment of cells collected on D0 significantly increased their activation to levels similar to D90. Conversely, treatment of D90 cells with rhTGFβ1 significantly decreased their activation to levels similar to D0.

Given the importance of TGFβ1 as a regulatory cytokine, we hypothesized that downregulation of TGFβ1 production might contribute to the trained immunity developed by monocytes in response to RZV administration. To test this hypothesis, we treated ex vivo purified monocyte and NK cell cocultures from 10 RZV recipients (demographics in **S4 Table**) with rhTGFβ1 or with the compound LY, which prevents the binding of TGFβ1 to its receptor and subsequent downstream signal transduction. Treatment of pre-vaccination cocultures with LY increased the activation of monocytes in response to VZV-rgE stimulation to levels comparable to VZV-rgE-stimulated cocultures of cells obtained on Day 90 post-vaccination (**Fig 6**). Conversely, treatment of cocultures of cells collected on Day 90 with rhTGFβ1 depressed VZV-rgE-induced monocyte activation to levels similar to those observed pre-vaccination.

## Discussion

The innate immune cell memory-like responses generated by vaccines may play an important role in protection against infections. For example, monocytes and NK cells whose functionality increased after BCG and Ad26-SIV vectored vaccine, respectively, were shown to contribute to protection against tuberculosis and simian immunodeficiency viral infection, respectively [27,60]. Here, we show that the highly efficacious RZV generates robust memory-like innate immune responses. In contrast, the less efficacious ZVL did not show evidence of memory-like immune response induction in innate immune cells, which is in agreement with previous studies showing weak activation of innate immune responses by ZVL [85]. RZV generated appreciable activation of NK cells, γδ T cells, monocytes and DC both in vivo and in vitro. This finding is supported by previous studies showing activation of NK and other innate cells in the draining lymph node of the site of AS01-adjuvanted vaccine inoculation [86,87]. After the 2^nd^ dose of RZV, in vitro gE-stimulated NK cells, DC and monocytes displayed persistently greater activation than before vaccination for up to 5 years, suggesting the development of durable memory-like responses. Using their rapid response capability, innate immune cells may contribute to initial control of the replication of reactivated VZV in the dorsal root ganglia or in the skin, ensuring that viral propagation does not progress to HZ. In fact, skin infiltration and a potential role of adaptive NK cells against HZ was described in the context of natural infection [42].

Innate immune cells acquire memory-like responses through epigenetic imprinting, which, in the case of short-lived myeloid cells, such as monocytes, DC, and macrophages, is maintained through chromatin modification of the hematopoietic stem and progenitor cells [47,60]. Our investigation of the monocyte epigenome of RZV recipients suggested decreased chromatin accessibility around *TGFβ1*, which was of particular interest because TGFβ is a regulatory cytokine that plays a key role in the differentiation of regulatory T cells and inhibits the activation and proliferation of other immune cells [88–90]. TGFβ was also shown to promote the differentiation of monocytes into myeloid-derived suppressor cells (MDSC), while neutralization or inhibition of TGFβ downstream signaling diverted the monocyte differentiation from MDSC to antigen-presenting DC [91]. Thus, downregulation of TGFβ1 expression in monocytes after vaccination, which we verified by transcriptomic and proteomic analyses, might increase the monocyte pro-inflammatory functions. We confirmed this hypothesis by showing that addition of rhTGFβ1 to VZV-gE-stimulated monocyte and NK cocultures significantly decreased the expression of monocyte and NK cell activation markers, while blocking TGFβ1 with the LY compound potentiated activation. These findings suggest a role for TGFβ1 downregulation in promoting durable increased activation in response to antigen stimulation in monocytes. This mechanism may be another avenue for trained immunity in monocytes via modulation of cytokine production, in addition to the previously described metabolic reprogramming [32] and transcription factor modulation [92].

In line with the establishment of trained immunity, we were also able to demonstrate increased heterologous innate immune responses in monocyte and NK cell cocultures stimulated with HCMV or HSV antigens. The generation of heterologous responses is in agreement with clinical observations associating RZV administration with protection against diseases caused by other viruses such as protection against COVID-19 [93]. However, we did not observe increased heterologous responses to R848 stimulation of TLR7 and TLR8 or to rhIL2. This finding is in agreement with previous studies showing decreased responses to TLR agonists in monocytes rendered hyperresponsive to antigens by the administration of adjuvanted hepatitis B vaccine [92]. The diversity of intracellular pathways activated by antigens and TLR agonists may contribute to this differential effect. Binding of TLRs 7 and 8 elicits downstream activation of interferon regulatory factors and NFκB [94,95]. In contrast, peptide binding to MHC activates Src kinases, and subsequent tyrosine phosphorylation is the main intracellular downstream signaling mechanism [96]. Moreover, T-cell or NK-cell binding to the MHC-peptide complexes on APC creates an immunologic synapse where CD80, CD86, and CD40 APC receptors bind to their stimulatory ligands on the effector cell, triggering additional activation signals in the APC [97,98] distinct from those generated by R848 binding to TLRs. The need for monocyte coculture with NK cells to reveal VZV-gE activation is consistent with the formation of immunologic synapses as an underlying mechanism. Previous studies showed that HCMV activates NK cells through the expression of peptides in the context of MHC class I E on monocytes, which are recognized by the NKG2C receptor on NK cells resulting in NK cell activation [99]. Moreover, NK cells express CD28 [100,101], which may bind to their CD80 and CD86 stimulatory ligands expressed by the monocytes. The importance of direct contact between monocytes and NK cells for NK cell activation was noted in previous studies [102].

Other genes that appeared to be downregulated by RZV in monocytes, *DNM2* and *ARAP1,* are involved in endocytosis and cell receptor activity [103–105], whereas the upregulated genes, *EFNA5* and *MGLL,* play a role in cell adhesion, extravasation, migration, [106] in lipid metabolism and synthesis of arachidonic acid, the precursor of prostaglandins and other inflammatory mediators [107,108]. Modifications in the expression of these genes may also contribute to protective memory-like immune responses against VZV and other viruses.

Although NK cells also developed epigenetic modifications after vaccination, the affected genes are not known to contribute to the immune response. HCMV adaptive NK cells were previously shown to undergo epigenetic modifications through DNA methylation^32^, which was not investigated in our study.

DCs also showed evidence of trained immunity in bulk PBMC cultures stimulated with VZV-gE. Moreover, the scRNA-seq analysis showed decreased *TGFβ1* transcription in monocyte-derived DC and cDC2, similar to our findings in monocytes. Although functional experiments using purified DC were not performed due to the limited number of DC in PBMC, the other findings suggested the development of trained immunity in DC post-RZV administration possibly through mechanisms similar to the mechanisms established in monocytes.

Our study had several limitations. We did not test samples obtained between 1 and 7 days after vaccination, which may have obscured additional increases in circulating activated innate and/or adaptive immune cells. It is also possible that a larger number of samples might be needed to detect significant increases in innate immune responses of ZVL recipients because pre-vaccination responses to VZV are greater than those to VZV-gE, while responses to VZV-gE after ZVL administration are very low as we previously demonstrated [55]. Collectively, these elements limited our evaluation of innate immune cell memory in ZVL recipients. Another limitation is that we did not investigate DNA methylation.

In conclusion, we showed that RZV induces memory-like responses in innate immune cells through modulation of cytokine gene expression, which may be a previously undescribed form of inducing trained immunity in this cell subset. The demonstration of memory-like innate cell responses against VZV-gE elicited by RZV warrants the examination of its role in protection against HZ.

## Supporting information

S1 Table Key resource table. (DOCX)

S2 Table Demographic characteristics of Figs 1 and 2 study population. (DOCX)

S3 Table Demographic characteristics of Fig 3 study population. (DOCX)

S4 Table Demographic characteristics of Fig 4 study population. (DOCX)

S5 Table Demographic characteristics of Fig 5 study population. (DOCX)

S1 Fig Schematic representation of the study design. Yellow highlights indicate the groups that contributed samples to this study.
(DOCX)

S2 Fig Gating strategy of the phenotypic unstimulated panels. **Panel A:** T cells and NK cells; **Panel B**: APC and B cells. **Panel C**: PDL-1 FMO.
(DOCX)

S3 Fig Gating strategy for the ex vivo stimulation experiments. **Panel A:** T cells and NK cells for the 1-year kinetics; **Panel B**: APC and B cells for the 1-year kinetics; **Panel C**: NK cells and APC for the 5-year kinetics.
(DOCX)

S4 Fig Correlation analysis of PDL-1 and CD83 expression on monocytes and DC. Data were derived from 10 RZV and 10 ZVL recipients. Asterisks indicate p < 0.01.
(DOCX)

S5 Fig Correlation analysis of in vivo activated innate immune cells with activated CD4+ T cells in RZV recipients. Data were derived from 10 RZV recipients whose in vivo immune cell activation kinetics are shown in **Fig 1**. Only the proportions of activated γδ T cells significantly correlated with the proportions of activated CD4 + T cells. Asterisks indicate p < 0.01 by Spearman correlation analysis.
(DOCX)

S6 Fig Innate and adaptive immune cell activation in response to ex vivo antigenic stimulation in ZVL recipients. Data were derived from 10 participants who received ZVL on Day 0. PBMC were stimulated overnight with VZV whole virus and mock control. The graphs show the frequency of activated cells in antigen-stimulated conditions after subtraction of mock control for each participant. p values for the comparison of pre-vaccination with post-vaccination results were calculated using Friedman test for repeated measures with FDR correction for multiple comparisons. Adaptive memory and innate memory-like immune responses were identified in B cells and CD4 + T cells in the 1^st^ year, DC up to Day 90 and in CD8 + T cells at Day 30 post-immunization.
(DOCX)

S7 Fig Correlation coefficients of CD4+ T cell and other immune cell responses to ex vivo stimulation. **Panel A:**. Correlation coefficients of gE-specific CD4 + T cell and other immune cell responses to ex vivo VZV-gE stimulation in RZV recipients. **Panel B:** Correlation coefficients of VZV-specific CD4 + T cell and other immune cell responses to ex vivo VZV stimulation in ZVL recipients. Asterisk indicates significant correlations: ** < 0.01; * < 0.05.
(DOCX)

S8 Fig Responses to TLR and rhIL2 stimulation of innate immune cells do not change after RZV administration. Data were derived from 14 RZV recipients who received 2 doses of vaccine at enrollment and 60 days later. The graphs show mean and SEM of the frequency of activated cells in R848- or rhIL2-stimulated conditions (as indicated on the graphs) after subtraction of medium control. p values were calculated by mixed effects analysis and corrected for multiple comparisons by FDR.
(DOCX)

S9 Fig CD14+ CD16+ CD56+ doublets include coupled monocytes and NK cells. Data were derived from 10 RZV recipients. **Panel A** shows the gating strategy for the identification of CD14 + CD16 + CD56 + doublets representing coupled monocytes and NK cells, and singlets representing highly activated monocytes. **Panel B** shows significantly higher proportions of CD14 + CD16 + CD56 + events among doublets compared to singlets demonstrating that the CD14 + CD16 + CD56 + doublets contained both monocytes and NK cells.
(DOCX)

S10 Fig Single cell RNA-seq clustering. **A left**, UMAP projection where each dot is a single cell, colored by lineage. **A right**, PBMC lineage markers matrix plot where hue represents Z-score expression of specific genes (x-axis) in different lineages (y-axis). **B left**, UMAP projection where each dot is a single cell within the myeloid compartment, colored by cell type. **B right**, Myeloid cell type markers matrix plot where hue represents Z-score expression of specific genes (x-axis) in different lineages (y-axis).
(DOCX)

S11 Fig Epigenetic modifications of NK cells obtained after RZV administration. The heatmap shows the 13 genes whose accessibility significantly changed in NK cells between Days 0 and 90 after vaccination (FDR p < 0.1) in 10 participants.
(DOCX)
