# Supplementary material for: The recombinant zoster vaccine induces trained immunity in monocytes through persistent downregulation of TGFβ
Source: PLoS Pathog. 2025 Dec 5;21(12):e1013759. doi: 10.1371/journal.ppat.1013759 (PMC12694829; doi:10.1371/journal.ppat.1013759)
Supplement: S1 Table — (DOCX) [file ppat.1013759.s001.docx]

|  |  |  | |
| --- | --- | --- | --- |
| REAGENT or RESOURCE | SOURCE | | IDENTIFIER |
| **Antibodies** | | | |
| CD14 PE-CF594 | BD Biosciences | Cat# 562335, RRID:AB_11153663 | |
| CD19 APC | BD Biosciences | Cat# 555415, RRID:AB_398597 | |
| CD20 Ax700 | BD Biosciences | Cat# 560631, RRID:AB_1727447 | |
| CD25 APCcy7 | BD Biosciences | Cat# 557753, RRID:AB_396859 | |
| CD3 Ax700 | BD Biosciences | Cat# 557943, RRID:AB_396952 | |
| CD3 PE-Cy7 | BD Biosciences | Cat# 557851, RRID:AB_396896 | |
| CD45RO APC-H7 | BD Biosciences | Cat# 561137, RRID:AB_10562194 | |
| CD45RO BV421 | BD Biosciences | Cat# 562649, RRID:AB_2737703 | |
| γδ TCR PE | BD Biosciences | Cat# 347907, RRID:AB_400359 | |
| HLA-DR APC-H7 | BD Biosciences | Cat# 561358, RRID:AB_10611876 | |
| PDL-1 BV421 | BD Biosciences | Cat# 568319 | |
| Brilliant Stain Buffer Plus | BD Biosciences | Cat# 566385, RRID:AB_2869761 | |
| CCR7 PE-Dazzle | BioLegend | Cat# 353235, RRID:AB_2563640 | |
| CD123 PerCPcy5.5 | BioLegend | Cat# 306016, RRID:AB_2264693 | |
| CD137 APC | BioLegend | Cat# 309809, RRID:AB_830671 | |
| CD137 PE | BioLegend | Cat# 309804, RRID:AB_314783 | |
| CD14 Ax488 | BioLegend | Cat# 325610, RRID:AB_830683 | |
| CD141 PE Dazzle | BioLegend | Cat# 344120, RRID:AB_2687144 | |
| CD16 FITC | BioLegend | Cat# 302006, RRID:AB_314206 | |
| CD19 Ax700 | BioLegend | Cat# 363034, RRID:AB_2616936 | |
| CD1c Ax700 | BioLegend | Cat# 331530, RRID:AB_2563657 | |
| CD25 BV785 | BioLegend | Cat# 302637, RRID:AB_11219197 | |
| CD38 BV421 | BioLegend | Cat# 356618, RRID:AB_2566231 | |
| CD56 PE-Cy7 | BioLegend | Cat# 318318, RRID:AB_604107 | |
| CD8 PerCPcy5.5 | BioLegend | Cat# 344710, RRID:AB_2044010 | |
| CD83 PE | BioLegend | Cat# 305308, RRID:AB_314516 | |
| HLA-DR APC | BioLegend | Cat# 361610, RRID:AB_2563200 | |
| HLA-DR APC-Cy7 | BioLegend | Cat# 307618, RRID:AB_493586 | |
| CD20 Ax700 | Biolegend | Cat# 302322, RRID:AB_493753 | |
| CD69 BV786 | Biolegend | Cat# 310932, RRID:AB_2561370 | |
| IFNγ APC | Biolegend | Cat# 506510, RRID:AB_315443 | |
| Human TruStain FcX (Fc Receptor Blocking Solution) | BioLegend | Cat# 422302, RRID:AB_2818986 | |
| True-Stain Monocyte Blocker | BioLegend | Cat# 426103 | |
| Zombie Aqua Viability Kit | BioLegend | Cat# 423102 | |
| Zombie Yellow Viability | BioLegend | Cat# 423104 | |
| **Virus Strains** | | | |
| VZV Oka | Weinberg lab | NA | |
| **Chemicals, peptides, and recombinant proteins** | | | |
| rgE | GSK | NA | |
| VZV-gE peptides | GSK | NA | |
| R848 | Mabtech | Cat# 3611-5X | |
| rhIL2 | Sigma | Cat# SRP3085-50UG | |
| LY2109761 | Sigma-Aldrich | Cat# SML2051 | |
| rhTGF-beta 1 protein | R&D Systems | Cat# 240-B-002/CF | |
| penicillin/streptomyocin | Gemini | Cat# 50-753-3040 | |
| L-glutamine | Gemini | Cat# 400-106-100 | |
| EDTA | Corning | Cat# 46-034-CI | |
| Brefeldin A | Sigma | Cat# B7651 | |
| **Other** | | | |
| 30% Bovine Serum Albumen (BSA) | Sigma | Cat# A9576-50ML | |
| AIM-V Medium | Gibco | Cat# 12-055-091 | |
| FBS | Gemini | Cat# 100-500-500 | |
| BD lysing Solution | BD Biosciences | Cat# 349202 | |
| BD Perm 2 | BD Biosciences | Cat# 340973 | |
| Hepes buffer | Corning | Cat# 25-060-CI | |
| ATAC-Seq Kit | Active Motif | Cat# 53150 | |
| EasySep Human Monocyte Enrichment Kit | Stemcell Technologies | Cat# 19059 | |
| EasySep Human NK Cell Enrichment Kit | Stemcell Technologies | Cat# 19059 | |
