# Supplementary material for: The recombinant zoster vaccine induces trained immunity in monocytes through persistent downregulation of TGFβ
Source: PLoS Pathog. 2025 Dec 5;21(12):e1013759. doi: 10.1371/journal.ppat.1013759 (PMC12694829; doi:10.1371/journal.ppat.1013759)
Supplement: S2 Table — (DOCX) [file ppat.1013759.s002.docx]

| Characteristic | ZVL  (N=10) | | RZV  (N=10) | | Total  (N=20) | |
| --- | --- | --- | --- | --- | --- | --- |
| Age: mean years (range) | 65 (51-79) | | 63 (50-77) | | 64 (50-79) | |
| Sex |  |  |  |  |  |  |
| Male | 4 | 40% | 4 | 40% | 8 | 40% |
| Female | 6 | 60% | 6 | 60% | 12 | 60% |
| Race |  |  |  |  |  |  |
| White | 10 | 100% | 9 | 90% | 19 | 95% |
| Black | 0 | 0% | 1 | 10% | 1 | 5% |
| Ethnicity |  |  |  |  |  |  |
| Hispanic | 1 | 10% | 0 | 0% | 1 | 5% |
| Non-Hispanic | 9 | 90% | 10 | 100% | 19 | 95% |

Abbreviations: ZVL, zoster vaccine live; RZV, recombinant zoster vaccine
