# Supplementary material for: The recombinant zoster vaccine induces trained immunity in monocytes through persistent downregulation of TGFβ
Source: PLoS Pathog. 2025 Dec 5;21(12):e1013759. doi: 10.1371/journal.ppat.1013759 (PMC12694829; doi:10.1371/journal.ppat.1013759)
Supplement: S5 Table — (DOCX) [file ppat.1013759.s005.docx]

| Characteristic | RZV  (N=20) | |
| --- | --- | --- |
| Age: mean years (range) | 61 (50-79) | |
| Sex |  |  |
| Male | 9 | 45% |
| Female | 11 | 55% |
| Race |  |  |
| White | 19 | 95% |
| Black | 1 | 5% |
| Ethnicity |  |  |
| Hispanic | 0 | 0% |
| Non-Hispanic | 20 | 100% |

Abbreviations: RZV, recombinant zoster vaccine
