# Supplementary figures and images for: The recombinant zoster vaccine induces trained immunity in monocytes through persistent downregulation of TGFβ
Source: PLoS Pathog. 2025 Dec 5;21(12):e1013759. doi: 10.1371/journal.ppat.1013759 (PMC12694829; doi:10.1371/journal.ppat.1013759)

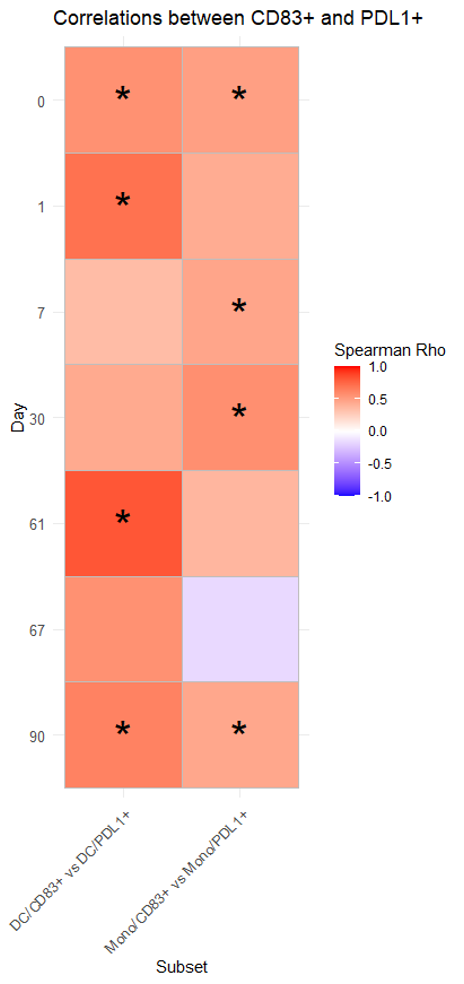

Supplement: S4 Fig — Asterisks indicate p < 0.01. (DOCX) [file ppat.1013759.s009.docx]

**
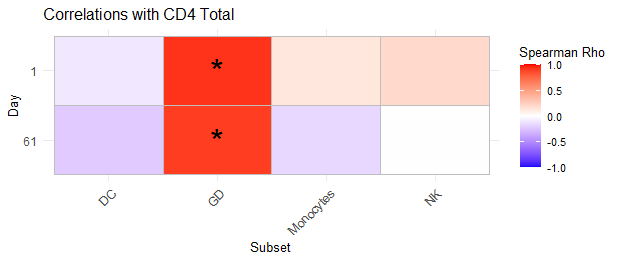
**

Supplement: S5 Fig — Data were derived from 10 RZV recipients whose in vivo immune cell activation kinetics are shown in Fig 1. Only the proportions of activated γδ T cells significantly correlated with the proportions of activated CD4 + T cells. Asterisks indicate p < 0.01 by Spearman correlation analysis. (DOCX) [file ppat.1013759.s010.docx]

**A**


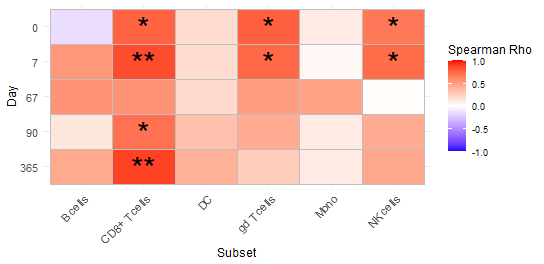


**B**

**
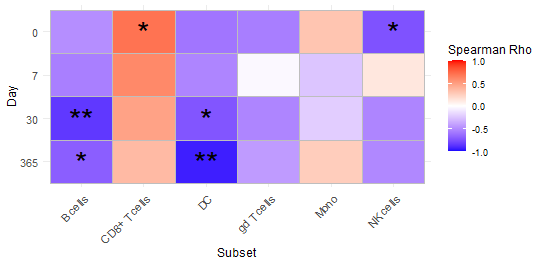
**

Supplement: S7 Fig — Panel A:. Correlation coefficients of gE-specific CD4 + T cell and other immune cell responses to ex vivo VZV-gE stimulation in RZV recipients. Panel B: Correlation coefficients of VZV-specific CD4 + T cell and other immune cell responses to ex vivo VZV stimulation in ZVL recipients. Asterisk indicates significant correlations: ** < 0.01; * < 0.05. (DOCX) [file ppat.1013759.s012.docx]

**
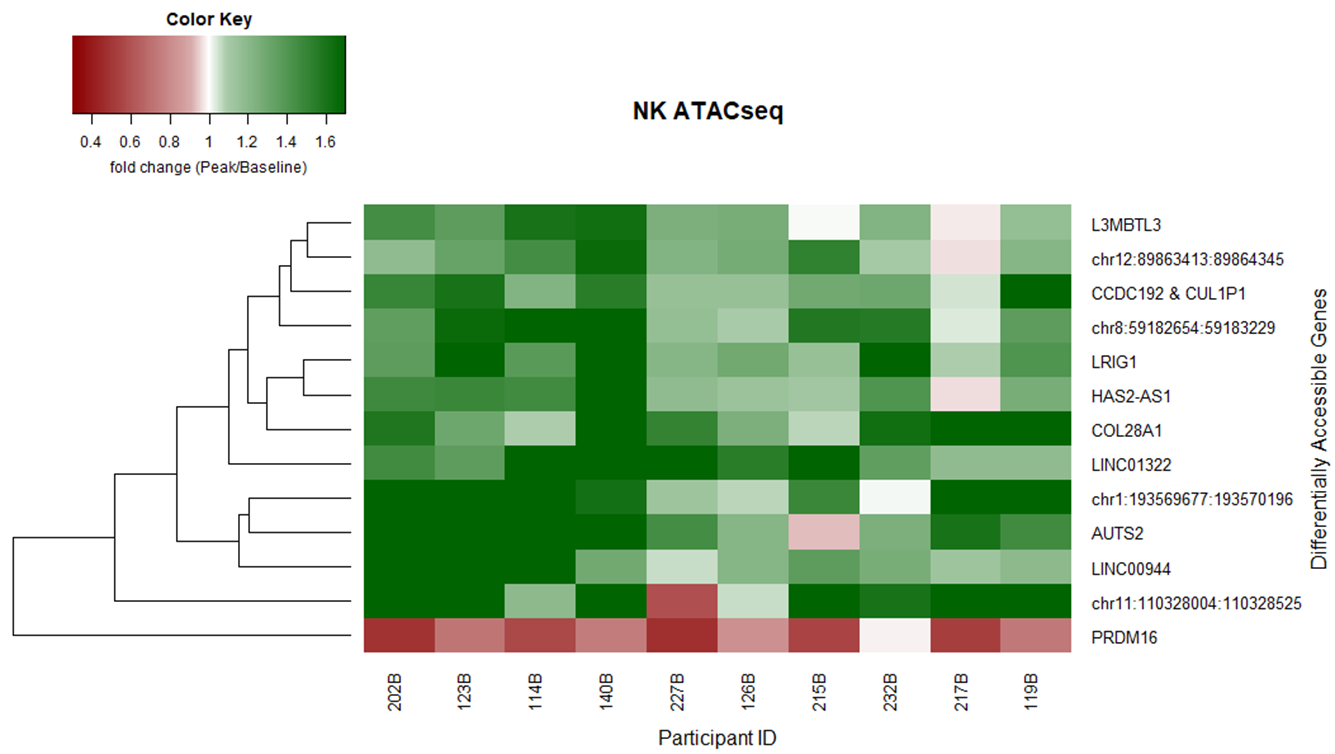
**

Supplement: S11 Fig — The heatmap shows the 13 genes whose accessibility significantly changed in NK cells between Days 0 and 90 after vaccination (FDR p < 0.1) in 10 participants. (DOCX) [file ppat.1013759.s016.docx]
