# Supplementary material for: The recombinant zoster vaccine induces trained immunity in monocytes through persistent downregulation of TGFβ
Source: PLoS Pathog. 2025 Dec 5;21(12):e1013759. doi: 10.1371/journal.ppat.1013759 (PMC12694829; doi:10.1371/journal.ppat.1013759)
Supplement: S9 Fig — Data were derived from 10 RZV recipients. Panel A shows the gating strategy for the identification of CD14 + CD16 + CD56 + doublets representing coupled monocytes and NK cells, and singlets representing highly activated monocytes. Panel B shows significantly higher proportions of CD14 + CD16 + CD56 + events among doublets compared to singlets demonstrating that the CD14 + CD16 + CD56 + doublets contained both monocytes and NK cells. (DOCX) [file ppat.1013759.s014.docx]

**B**

**A**

**A**
