# Supplementary material for: The recombinant zoster vaccine induces trained immunity in monocytes through persistent downregulation of TGFβ
Source: PLoS Pathog. 2025 Dec 5;21(12):e1013759. doi: 10.1371/journal.ppat.1013759 (PMC12694829; doi:10.1371/journal.ppat.1013759)
Supplement: Supplementary file 8 [file ppat.1013759.s006.docx]

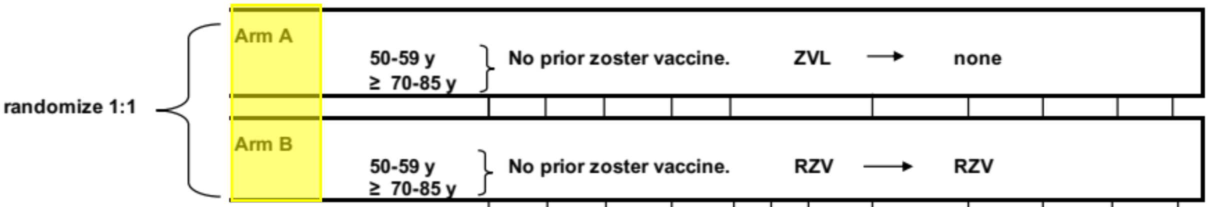

Visit #	1	2	3	4	5	6	7	8	9	10	11	12
Day / [year]	0	1	7	30	60	61	67	90	1 y	2 y	4 y	5 y
Vaccine	+				+							
Blood Draw	+	+	+	+				+	+	+	+	+
Blood Draw (Arm B only)						+	+					
Saliva (Arm A only)	+	+										

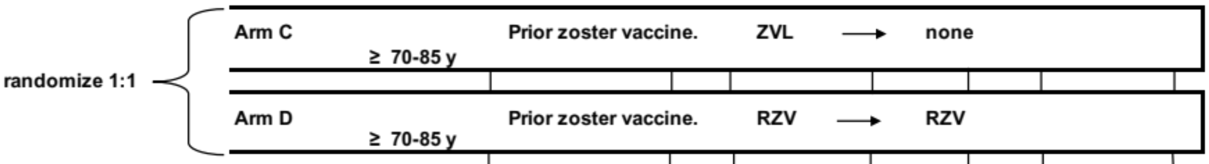

Visit #	1	2	3	4	5	6	7
Day / [year]	0	30	60	90	1 y	2 y	5 y
Vaccine	+		+				
Blood Draw	+	+		+	+	+	+
