# Supplementary material for: The recombinant zoster vaccine induces trained immunity in monocytes through persistent downregulation of TGFβ
Source: PLoS Pathog. 2025 Dec 5;21(12):e1013759. doi: 10.1371/journal.ppat.1013759 (PMC12694829; doi:10.1371/journal.ppat.1013759)

DC PDL1+

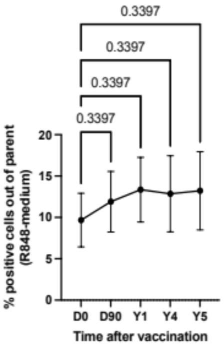

Mono PDL1+

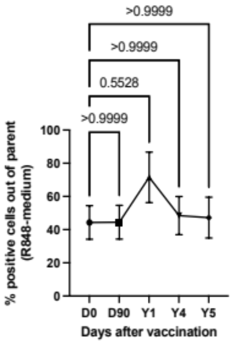

NK CD25+CD137+

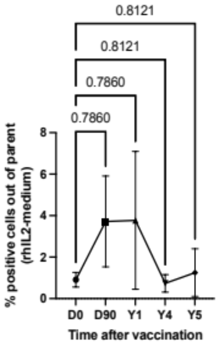

Supplement: Supplementary file 10 [file ppat.1013759.s013.docx]
